# Efficacy and safety of cadonilimab combined with chemotherapy for gastric or gastroesophageal junction adenocarcinoma: a single-arm meta-analysis

**DOI:** 10.3389/fimmu.2026.1693179

**Published:** 2026-02-18

**Authors:** Likun Yang, Jiaxin Li, Ximo Wang, Xiangyang Yu, Zhengcun Pei

**Affiliations:** 1 Medical School, Tianjin University, Tianjin, China; 2 Tianjin Third Central Hospital, Tianjin, China; 3 Tianjin Medical University Cancer Institute and Hospital, Tianjin, China

**Keywords:** bispecific antibody, cadonilimab, gastric cancer, gastroesophageal junction cancer, meta-analysis

## Abstract

**Background:**

Immune checkpoint inhibitors (ICIs) combined with chemotherapy have become an important area of clinical investigation in the gastric cancer (GC) and gastroesophageal junction cancer (GEJC). In recent years, bispecific antibody therapies have been increasingly explored, including cadonilimab, a programmed death-1/cytotoxic T-lymphocyte–associated antigen 4 (PD-1/CTLA-4) bispecific antibody. Several phase II studies have reported early signals of antitumor activity with cadonilimab, prompting clinical interest in this strategy. However, treatment outcomes vary across studies, and a systematic assessment of the efficacy and safety of cadonilimab plus chemotherapy remains limited. This study aimed to evaluate the efficacy and safety profile of cadonilimab in combination with chemotherapy for gastric adenocarcinoma and gastroesophageal junction adenocarcinoma (G/GEJ adenocarcinoma) through a single-arm meta-analysis.

**Methods:**

A systematic search was conducted in PubMed, Embase, the Cochrane Library, Web of Science, and ClinicalTrials.gov to identify eligible studies published up to July 20, 2025. A single-arm meta-analysis was performed to pool and evaluate the objective response rate (ORR), disease control rate (DCR), progression-free survival (PFS), pathological complete response (pCR), and the incidence of grade ≥3 treatment-related adverse events (TRAEs).

**Results:**

A total of four clinical studies involving 182 patients were included. The pooled estimates were an ORR of 57% (95% CI: 48%–66%), a DCR of 97% (95% CI: 84%–100%), a pCR of 23% (95% CI: 12%–35%), and a median PFS of 7.98 months (95% CI: 6.38–9.57). The overall incidence of grade ≥3 TRAEs was 35% (95% CI: 7%–63%). In subgroup analyses, the incidence of grade ≥3 TRAEs appeared lower in the neoadjuvant setting than in the first-line setting (29% [95% CI: 28%–40%] vs 53% [95% CI: 45%–60%]). The most frequently reported adverse events included nausea (72%), vomiting (60%), neutropenia (51%), and leukopenia (45%).

**Conclusions:**

Based on available early-phase evidence, cadonilimab plus chemotherapy showed antitumor activity with a manageable safety profile in G/GEJ adenocarcinoma. Further large-scale, high-quality randomized controlled trials are needed to validate these findings and define the optimal treatment strategy.

**Systematic Review Registration:**

https://www.crd.york.ac.uk/PROSPERO, identifier CRD420251089855.

## 1 Introduction

GC and GEJC remain a substantial global health burden. In 2022, more than 968,000 new cases and nearly 660,000 deaths were reported worldwide, ranking fifth in cancer incidence and fourth in cancer-related mortality (1, 2). East Asia, particularly China, bears a disproportionately high disease burden, contributing approximately 42% of new global cases and 45% of GC-related deaths (3, 4). Due to the lack of specific symptoms in early stages, over 80% of patients are diagnosed at a locally advanced or metastatic stage, thereby losing the opportunity for curative surgery. For resectable G/GEJ adenocarcinoma, perioperative chemotherapy is commonly used, and platinum-based neoadjuvant or adjuvant regimens are widely recommended to improve resectability and reduce recurrence risk. For advanced or metastatic disease, platinum plus fluoropyrimidine chemotherapy, either alone or combined with human epidermal growth factor receptor 2 (HER2) or vascular endothelial growth factor receptor (VEGFR)–targeted therapy, remains the standard first-line systemic treatment strategy (5–8). However, the efficacy of these approaches remains limited across both perioperative and advanced disease settings. A considerable proportion of patients experience early postoperative recurrence, and the median overall survival (OS) for advanced cases remains under one year (9). Therefore, there is an urgent need to develop more effective treatment strategies to improve outcomes across different disease stages.

In recent years, ICIs have shown clinical benefit in multiple solid tumors, thereby supporting their increasing evaluation across different treatment settings for G/GEJ adenocarcinoma (10–13). The most widely studied immune checkpoints include PD-1, programmed death-ligand 1 (PD-L1), and CTLA-4. By blocking these inhibitory pathways, ICIs can relieve T-cell suppression within the tumor microenvironment and enhance antitumor immune activity, potentially inducing more durable immune responses. Several phase III clinical trials (e.g., CheckMate 649, KEYNOTE-859, ORIENT-16, GEMSTONE-303 and COMPASSION-15) have reported improved OS and PFS with PD-1 inhibitors plus chemotherapy in patients with HER2-negative advanced G/GEJ adenocarcinoma, supporting immunochemotherapy as a first-line treatment option. With accumulating evidence, the potential role of ICIs in the perioperative setting has also received increasing attention. Nevertheless, the long-term efficacy of immunotherapy remains suboptimal, with 5-year survival rates generally below 20% (14–17). In gastric adenocarcinoma, high expression levels of PD-L1 (approximately 40%) and CTLA-4 (approximately 85%) have been associated with poor prognosis, providing a biological rationale for dual immune checkpoint blockade (18–21). Building on this immunological foundation, combined blockade of PD-1 and CTLA-4 (e.g., nivolumab plus ipilimumab) has shown enhanced antitumor activity and clinical benefit in multiple solid tumors, further supporting the rationale for dual immune checkpoint blockade (22–28).

Cadonilimab (AK104) is a bispecific antibody targeting PD-1 and CTLA-4, with an optimized Fc domain to enhance antitumor efficacy while minimizing immune-related toxicity. Preclinical models and early-phase clinical studies in solid tumors have suggested a manageable safety profile and preliminary antitumor activity (29, 30). In the phase I/II COMPASSION-03 study, cadonilimab was generally well tolerated in patients with advanced solid tumors, and clinical responses were observed (31–34). Based on these findings, cadonilimab has been approved in China for the treatment of recurrent or metastatic cervical cancer (35). In gastric cancer, cadonilimab has also been evaluated in the COMPASSION trial series. Notably, the phase III AK104-302 (COMPASSION-15) trial reported an ORR of 65.2% and a median OS of approximately 15 months in patients with advanced G/GEJ adenocarcinoma treated with cadonilimab plus chemotherapy (36). As a dual immune checkpoint inhibitor, cadonilimab may enhance antitumor immune activity through coordinated blockade of PD-1 and CTLA-4 pathways, potentially improving treatment response while maintaining a manageable safety profile in G/GEJ adenocarcinoma, which is often characterized by an immunosuppressive tumor microenvironment. Against this background, we conducted a systematic single-arm meta-analysis integrating available phase II clinical trial data to comprehensively evaluate the efficacy and safety of cadonilimab in combination with chemotherapy for G/GEJ adenocarcinoma across different treatment settings, including both perioperative and advanced disease stages. This study aims to provide a quantitative synthesis of available evidence regarding the efficacy and safety of cadonilimab plus chemotherapy in G/GEJ adenocarcinoma.

## 2 Methods

### 2.1 Protocol and reporting guidelines

This study was registered in the International Prospective Register of Systematic Reviews (registration number: CRD420251089855) and was conducted and reported in strict accordance with the 2020 updated guidelines of the Preferred Reporting Items for Systematic Reviews and Meta-Analyses (PRISMA) statement (37).

### 2.2 Information sources and search strategy

A comprehensive literature search was conducted using PubMed, Embase, the Cochrane Library, Web of Science, and ClinicalTrials.gov to identify all relevant clinical studies published up to July 20, 2025. The search strategy incorporated both Medical Subject Headings and free-text terms; detailed search algorithms are provided in **Supplementary Table S1**. To enhance the completeness and sensitivity of study inclusion, we also manually reviewed supplementary materials and the reference lists of all included articles. Two reviewers independently screened the records according to predefined inclusion and exclusion criteria, with literature management performed using EndNote software (EndNote 21.5, Clarivate, Philadelphia, PA, USA). Any disagreements were resolved by consultation with a third reviewer. The screening process was conducted in two stages: initial screening of titles and abstracts, followed by full-text review of potentially eligible studies.

### 2.3 Selection criteria

Studies were eligible for inclusion if they met the following criteria: 1) Population: Patients with a confirmed diagnosis of untreated G/GEJ adenocarcinoma; 2) Intervention: Treatment with cadonilimab in combination with chemotherapy; 3) Study design: Prospective clinical trials (phase I, II, or III), retrospective cohort studies, or real-world evidence studies; 4) Outcomes: Studies reporting at least one clinically relevant outcome related to efficacy or safety, including but not limited to ORR, DCR, PFS, OS, TRAEs or immune-related adverse events (irAEs).

The following exclusion criteria were applied: 1) *In vitro* studies, animal experiments, review articles, meta-analyses, case reports, editorials, or conference commentaries; 2) Studies from which data relevant to the primary outcomes could not be extracted; 3) Publications not written in English.

### 2.4 Data extraction and quality assessment

Two reviewers independently performed data extraction and quality assessment. Discrepancies were resolved by consultation with a third reviewer. The following information was collected whenever available: trial registration number, first author, publication year, study period, sample size, treatment regimen, Eastern Cooperative Oncology Group Performance Status (ECOG PS), PD-L1 Combined Positive Score (CPS), primary tumor location, metastatic sites, and key clinical efficacy and safety outcomes, including ORR, DCR, OS, PFS, TRAEs and irAEs. Additionally, details related to study design were extracted to assess potential risk of bias.

For non-randomized studies, we systematically evaluated study quality using the Risk of Bias in Non-randomized Studies of Interventions (ROBINS-I) tool, which addresses seven domains of bias: confounding, selection of participants, classification of interventions, deviations from intended interventions, missing data, measurement of outcomes, and selection of the reported result (38). Each domain was rated as having low, moderate, or high risk of bias. All assessments were conducted independently by two reviewers, and inter-rater agreement was measured using Cohen’s kappa statistic.

### 2.5 Statistical analysis

Pooled effect estimates were calculated using either fixed-effects or random-effects models, depending on the degree of statistical and clinical heterogeneity. Heterogeneity was assessed using Cochran’s Q test and the I² statistic. Significant heterogeneity was defined as I² > 50% and a p-value < 0.10 for the Q test. When statistical or clinical heterogeneity was present, a random-effects model was applied; when heterogeneity across studies was low, a fixed-effects model was used. To evaluate treatment efficacy, pooled estimates of ORR, DCR and PFS were calculated. For safety assessment, the incidence of TRAEs and irAEs was extracted and analyzed across studies to provide a comprehensive safety profile. Publication bias was assessed using funnel plots. Sensitivity analyses were performed using the leave-one-out method to examine the robustness of the pooled estimates. All statistical analyses were conducted using Stata/MP 18.0 (StataCorp LLC, College Station, TX, USA) and R software (version 4.4.1).

## 3 Results

### 3.1 Study selection

**Figure 1:**
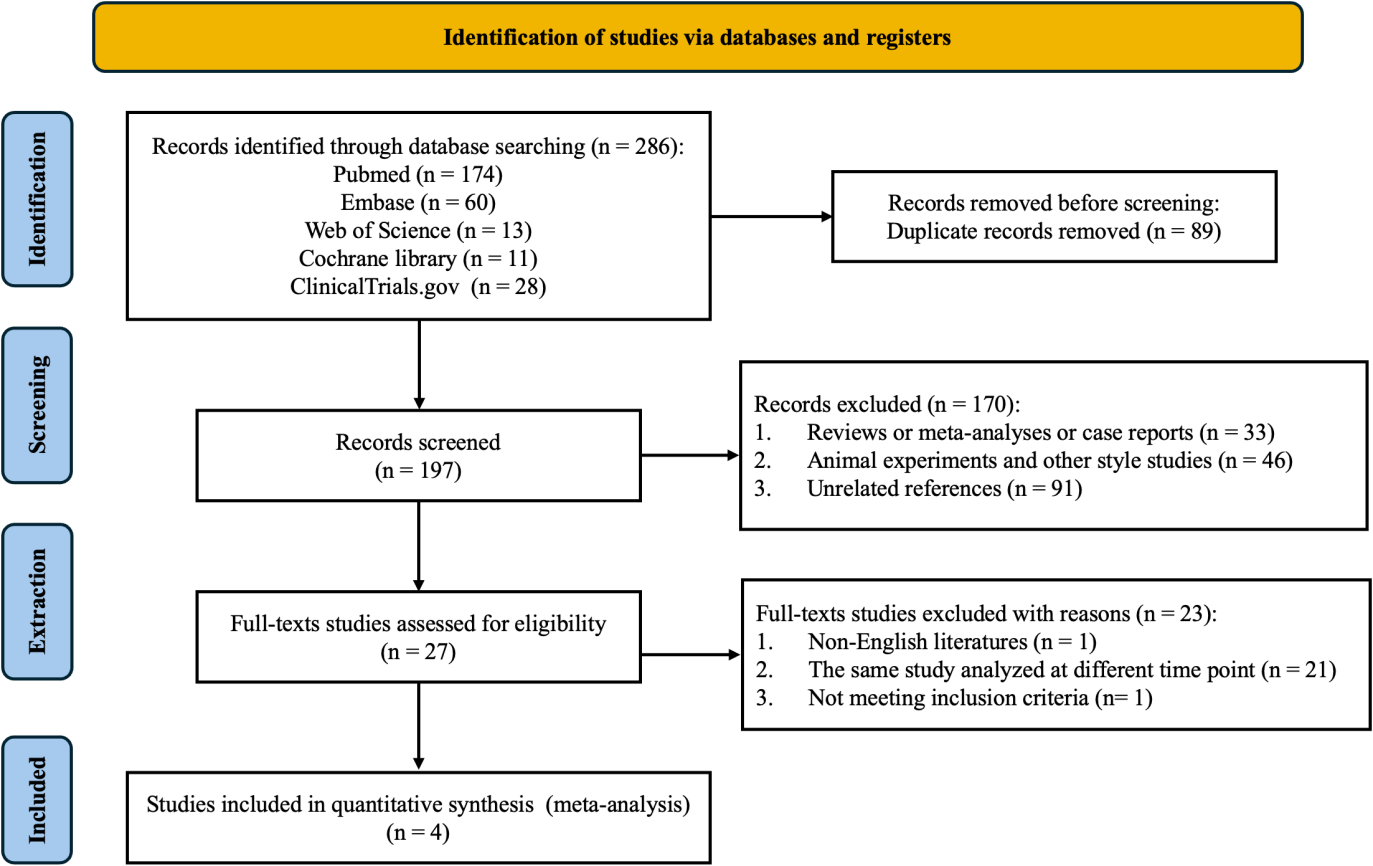
PRISMA flow diagram of study selection.

A total of 286 potentially relevant studies were initially retrieved from five databases: PubMed (n = 174), Embase (n = 60), Web of Science (n = 13), the Cochrane Library (n = 11), and ClinicalTrials.gov (n = 28). After removing duplicates, 197 unique records remained. Following title and abstract screening, 27 studies were selected for full-text review. Ultimately, four studies met the inclusion criteria and were included in the final meta-analysis (39–42). The study selection process is illustrated in **Figure 1**, and the key characteristics of the included studies are summarized in **Table 1**.

**Table 1:** Overview of the included trials.

First author	Clinicaltrial No.	Phase study center	Year of publication	Study period	Inclusion criteria	Interventions	Reported outcomes
B. Long	ChiCTR2200066893	II Multicenter	Mar 2025	Dec 2022- Dec 2023	Locally advanced, treatment-naive GC/GEJC (cT3/4, N+, M0), HER2-negetive	Cadonilimab 10 mg/kg Q3W + FLOT + radical D2 gastrectomy	pCR, MPR, DCR, R0,TRAEs
P. Zhang	NCT05948449	Ib/II Single center	Sep 2024	Sep 2023- Apr 2024	Locally advanced GC/GEJC (cT3-4a N+ M0, CY0, P0)	Cadonilimab + SOX + radical D2 gastrectomy	pCR, R0, MPR, DFS, OS, TRAEs
X. Gao	ChiCTR20182027	II Single center	May 2024	May2019-Aug 2021	Unresectable advanced or metastatic GC/GEJC, HER2-negetive	Cadonilimab 6 mg/kg Q2W or 10 mg/kg Q3W + mXelox/Xelox	ORR, DCR, DoR, TTR, PFS, OS, TRAEs
Q. Xu	NR	II Single center	Nov 2023	Aug 2022- Aug 2023	Advanced GC/GEJC with PD-L1 CPS ≥ 5	Cadonilimab 10 mg/kg Q3W + SOX/XELOX or Cadonilimab 6 mg/kg Q2W + FOLFOX	ORR, DCR, PFS, OS, TRAEs

GC, gastric cancer; GEJC, gastroesophageal junction cancer; CPS, combined positive score; pCR, pathological complete response; MPR, major pathological response; DCR, disease control rate; R0, complete (R0) resection; ORR, objective response rate; DoR, duration of response; TTR, time to response; DFS, disease-free survival; PFS, progression-free survival; OS, overall survival; TRAEs, treatment-related adverse events; XELOX: oxaliplatin 130 mg/m² on day 1 + capecitabine 1000 mg/m² twice daily on days 1-14; SOX, oxaliplatin + S-1; FLOT, docetaxel 50 mg/m² + oxaliplatin 85 mg/m² + leucovorin 200 mg/m² + 5-fluorouracil 2600 mg/m² day1 q2w; FOLFOX: oxaliplatin + leucovorin + 5-fluorouracil; cTNM, clinical TNM stage based on the 8th edition of the AJCC staging system. NR, not reported.

### 3.2 Baseline characteristics

A total of four prospective clinical trials were included, encompassing 182 patients with G/GEJ adenocarcinoma. The median age across studies ranged from 55.5 years (30–71) to 62.7 years (29–75), and the proportion of male patients varied between 50.0% and 89.5%. Three studies reported the proportion of patients with an ECOG PS of 0–1, which ranged from 72.7% to 100%. Additionally, three studies provided data on the primary tumor site, with GC accounting for 71.1% to 87.2% and GEJC accounting for 12.8% to 28.9%. All included studies were phase II clinical trials; one was a multicenter trial, while the others were conducted at single centers. All patients received cadonilimab in combination with chemotherapy, including regimens such as FLOT, SOX, XELOX, modified XELOX (mXELOX), or FOLFOX. Detailed characteristics of each study are presented in **Table 2**.

**Table 2:** Baseline characteristics of the included studies.

First author	Sample size	Gender (male/female)	Median age (range,years)	ECOG (0 vs.1, %)	Tumor location (GC vs. GEJC,%)	Tumor status	Metastatic sites
B. Long	38	34/4	57.0 (38-74)	63.2% vs. 36.8%	71.1% vs. 28.9%	NR	NR
P. Zhang	24	NR	58.5 (27-72)	NR	79.2% vs. 20.8%	NR	NR
X. Gao	98	66/32	62.7 (29-75)	37.2% vs. 62.8%	87.2% vs. 12.8%	Locally advanced unresectable disease (5.3%), metastatic disease (80.9%), recurrent disease (13.8%)	Liver metastasis (44.7%), Peritoneal metastasis (8.5%), Lung metastasis (12.8%)
Q. Xu	22	11/11	55.5 (30-71)	72.7%(total)	NR	NR	Liver metastasis (27.3%)

First author	Her-2 Status	PD-L1 CPS Status	Lauren	Borrmann	MSI	Clinical T stage	Clinical N stage
B. Long	negative	CPS≥5 (34.2%)	Intestinal (26.3%) Diffused (36.8%) Mixed (36.8%)	II (13.2%) III (71.1%) IV (15.8%)	MSI-H (5.2%) MSS (94.7%)	cT3 (39.5%) cT4 (60.5%)	cN1 (47.4%) cN2 (39.5%) cN3 (13.2%)
P. Zhang	NR	NR	NR	NR	NR	cT3 (29.2%) cT4 (70.8%)	NR
X. Gao	negative	CPS≥5 (14.9%)	Intestinal (42.6%) Diffused (29.8%) Mixed (25.5%) Unknown (2.1%)	NR	NR	NR	NR
Q. Xu	NR	NR	NR	NR	NR	NR	NR

ECOG, Eastern Cooperative Oncology Group; MSI-H, microsatellite instability-high; MSS, microsatellite stable; NR, not reported.

### 3.3 Quality assessment

In the quality assessment using the ROBINS-I tool, the overall risk of bias among the included studies was low. Specifically, none of the four studies demonstrated serious or critical bias; three studies were rated as having a moderate risk of bias, and one study was rated as low risk (**Supplementary Figure S1**). To further evaluate inter-rater consistency, we calculated agreement across eight scoring domains, including seven bias domains and one overall risk-of-bias judgment. Results showed that, except for “confounding” and “missing data,” which had Cohen’s kappa values of 0.5 (95% CI: –0.235 to 1.000), all other domains yielded perfect agreement with kappa values of 1.000 (95% CI: 1.000 to 1.000), indicating a high level of concordance between the two reviewers (**Supplementary Table S2**).

### 3.4 Meta-analysis results

#### 3.4.1 Tumor response

**Figure 2:**
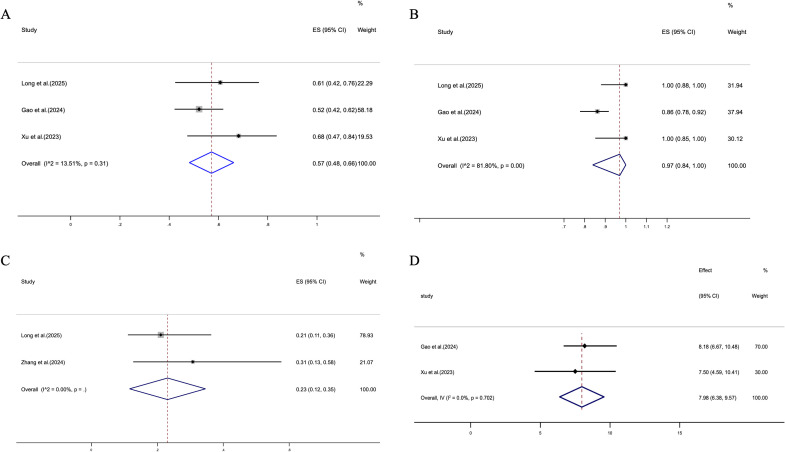
Forest plots summarizing the efficacy outcomes of Cadonilimab combined with chemotherapy in patients with G/GEJ adenocarcinoma. **(A)** ORR; **(B)** DCR; **(C)** pCR; **(D)** Median PFS.

All included studies reported efficacy outcomes of cadonilimab combined with chemotherapy in the treatment of G/GEJ adenocarcinoma. Three studies reported ORR, with low heterogeneity observed prior to pooling (I² = 13.5%, p = 0.31); therefore, a fixed-effects model was applied, yielding a pooled ORR of 57% (95% CI: 48%–66%) (**Figure 2A**). Another three studies reported DCR, which exhibited substantial heterogeneity (I² = 81.8%, p < 0.01); hence, a random-effects model was employed, resulting in a pooled DCR of 97% (95% CI: 84%–100%) (**Figure 2B**). In addition, two studies provided data on pCR. Given the negligible heterogeneity (I² = 0.0%) and the limited number of studies, a reliable p-value from the Q test could not be estimated. A fixed-effects model was therefore adopted, and the pooled pCR was 23% (95% CI: 12%–35%) (**Figure 2C**). Overall, the pooled results indicate that cadonilimab plus chemotherapy is associated with measurable tumor responses in G/GEJ adenocarcinoma.

#### 3.4.2 Survival analysis

Among the included studies, two reported PFS data. A fixed-effects model was applied (I² = 0.0%, p = 0.702), yielding a pooled median PFS of 7.98 months (95% CI: 6.38–9.57), as shown in **Figure 2D**. Regarding OS, two studies had insufficient data and one did not report OS, making it infeasible to perform a meta-analysis for this endpoint at this time.

#### 3.4.3 Safety analysis

**Figure 3:**
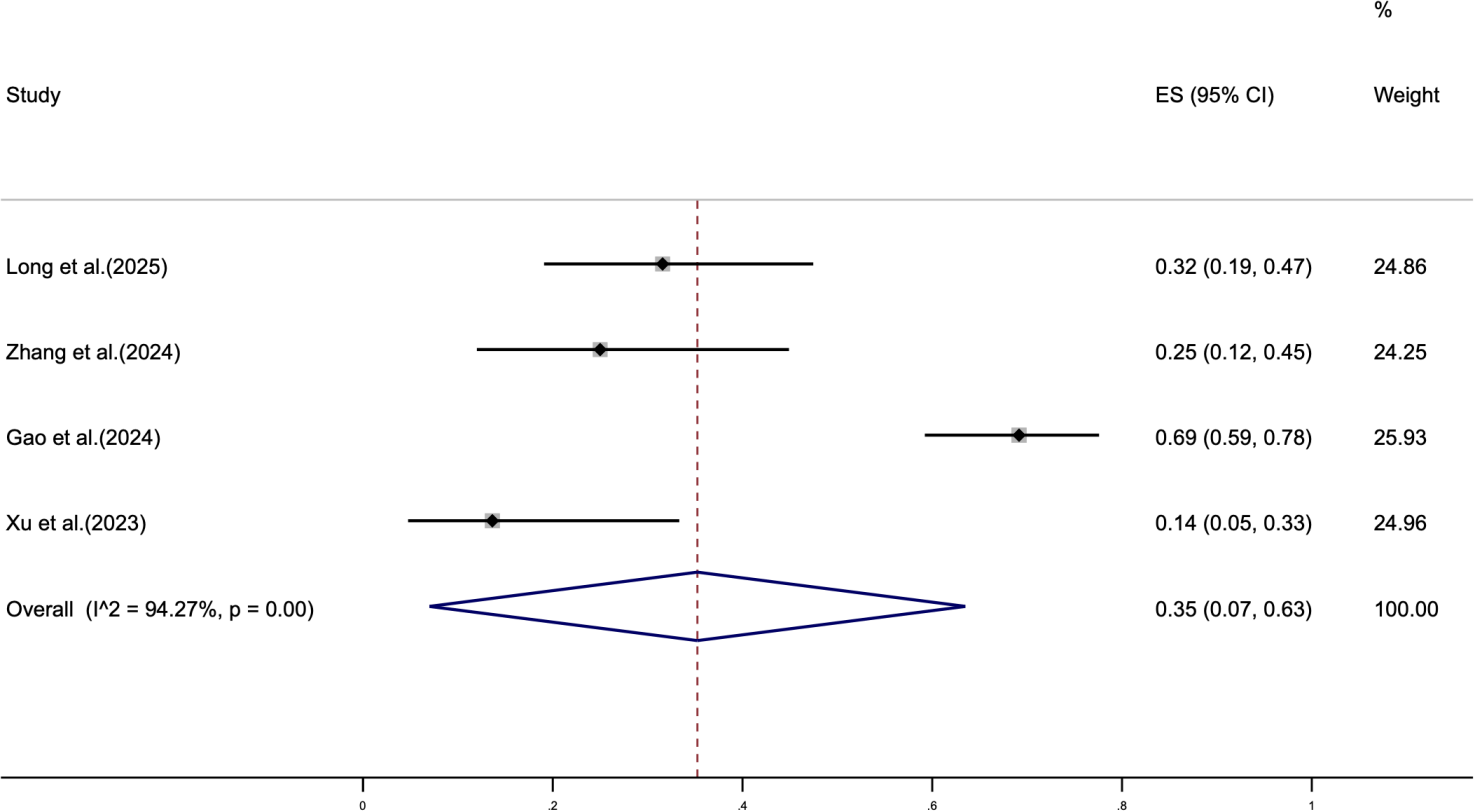
Forest plot showing the pooled incidence of grade 3 TRAEs in patients treated with Cadonilimab combined with chemotherapy.

We analyzed the most common grade 3 TRAEs associated with cadonilimab in patients with G/GEJ adenocarcinoma, with a pooled incidence of 35% (95% CI: 7%–63%). No grade 4 or 5 TRAEs were reported in any of the included studies (**Figure 3**). The most frequently observed adverse events were nausea, vomiting, neutropenia, and leukopenia, with incidences of 72% (95% CI: 17%–100%), 60% (95% CI: 21%–99%), 51% (95% CI: 38%–64%), and 45% (95% CI: 26%–65%), respectively (**Table 3**, **Supplementary Figure S2**). Additionally, adverse events reported in only a single study are summarized in **Supplementary Table S3**.

**Table 3:** Summary of pooled incidence and heterogeneity of TRAEs.

TRAEs	Study (n)	I^2^	P	Rate, 95%CI
Grade3	4	94.27%	<0.01	0.35(0.07, 0.63)
Nausea	3	92.76%	<0.01	0.72(0.17, 1.00)
Vomiting	3	96.66%	<0.01	0.60(0.21, 0.99)
Neutropenia	3	57.27%	0.10	0.51(0.38, 0.64)
Leukopenia	3	82.32%	<0.01	0.45(0.26, 0.65)
Anemia	3	55.09%	0.11	0.43(0.30, 0.55)
Thrombocytopenia	3	97.09%	<0.01	0.36(-0.03, 0.75)
ALT or AST increase	3	60.62%	0.08	0.30(0.18, 0.42)
Diarrhea	3	88.63%	<0.01	0.25(0.04, 0.45)
Hypothyroidism	3	60.49%	0.08	0.10(0.03, 0.18)
Fatigue	2	0.00%	<0.01	0.81(0.72, 0.91)
Rash	2	0.00%	<0.01	0.28(0.21, 0.35)
Fever	2	0.00%	<0.01	0.25(0.18, 0.33)
Hyperthyroidism	2	0.00%	<0.01	0.11(0.06, 0.16)
Hyperglycemia	2	0.00%	<0.01	0.04(-0.02, 0.10)

### 3.5 Subgroup analysis

**Figure 4:**
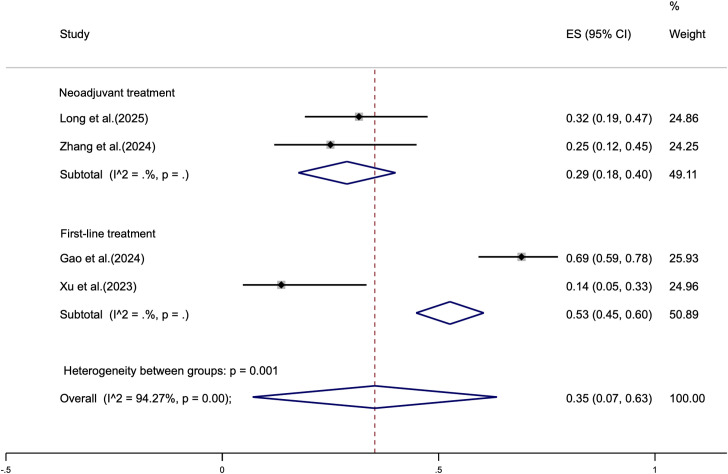
Forest plot of the pooled incidence of Grade 3 TRAEs stratified by treatment setting: neoadjuvant versus first-line therapy.

To further investigate the safety profile of cadonilimab combined with chemotherapy across different treatment settings, a subgroup analysis was performed based on treatment intent, categorizing the included studies into neoadjuvant and first-line advanced treatment groups. The pooled incidence of grade 3 TRAEs was 29% (95% CI: 28%–40%) in the neoadjuvant group and 53% (95% CI: 45%–60%) in the first-line advanced treatment group, with a statistically significant difference between the two (p < 0.01) (**Figure 4**). In the advanced treatment group, the most common adverse events leading to treatment interruption or dose adjustment included nausea, vomiting, neutropenia, and thrombocytopenia. Additionally, three treatment-related deaths were reported in this group, attributed to interstitial pneumonia, suspected COVID-19 infection, and myocarditis, respectively. In contrast, only one case of Clavien-Dindo grade 3 preoperative ascites was reported in the neoadjuvant group, which resolved uneventfully after paracentesis. No significant postoperative complications or treatment-related deaths were observed in this cohort. It should be noted that this subgroup analysis is limited by the small sample size, and the findings warrant further validation in large-scale prospective studies.

### 3.6 Risk of bias and sensitivity analysis

A leave-one-out sensitivity analysis was performed to evaluate the robustness of the pooled outcomes. The results indicated that the exclusion of any single study did not substantially alter the overall effect size or its 95% confidence interval, suggesting that the findings of this meta-analysis are generally stable and reliable.

### 3.7 Publication bias

Given the limited number of included studies, statistical tests such as Egger’s or Begg’s were not conducted to formally assess publication bias. Nevertheless, a funnel plot was generated to visually examine the potential presence of publication bias (**Supplementary Figure S3**).

## 4 Discussion

This single-arm meta-analysis synthesized the currently available phase II evidence on cadonilimab combined with chemotherapy in patients with G/GEJ adenocarcinoma, integrating data across perioperative and advanced treatment settings. The pooled estimates suggested measurable antitumor activity, as reflected by ORR, DCR, pCR and median PFS, supporting further investigation of this dual checkpoint blockade strategy in G/GEJ adenocarcinoma. From a broader clinical perspective, our findings are conceptually aligned with the treatment paradigm established by large phase III trials of PD-1 inhibitors plus chemotherapy in advanced HER2-negative disease, such as CheckMate 649 (14) and ATTRACTION-04 (43), in which immunochemotherapy has been associated with improved survival outcomes. Cadonilimab is a bispecific antibody designed to simultaneously target PD-1 and CTLA-4, and available studies have reported antitumor activity with a manageable immune-related toxicity profile across multiple tumor types (44, 45). Recent phase III evidence (COMPASSION-15) further supports the clinical activity of cadonilimab plus chemotherapy in advanced HER2-negative G/GEJ adenocarcinoma (36). Mechanistically, tumor cells may evade immune surveillance through concurrent upregulation of PD-1 and CTLA-4 pathways: PD-1 predominantly contributes to effector T-cell exhaustion, whereas CTLA-4 can suppress early T-cell priming and promote regulatory T-cell (Treg) infiltration, collectively dampening antitumor immunity (46–49). In certain contexts, PD-L1 blockade alone may inadvertently enhance Treg activity and immune tolerance; while additional CTLA-4 inhibition could help counterbalance this effect and reinforce immune activation (50, 51). Consistent with this framework, dual PD-1/CTLA-4 blockade has been proposed to overcome immune dysfunction and prolong antitumor responses, and has shown enhanced immunostimulatory potential across multiple solid tumors (52–54). Preclinical studies further suggest that cadonilimab may induce PD-1 and CTLA-4 receptor internalization, increase key cytokine production (e.g., IL-2 and IFN-γ), and reduce Treg infiltration (55), thereby contributing to improved T-cell function within the tumor environment. This may be particularly relevant to G/GEJ adenocarcinoma, which often exhibits limited baseline immune activation, and provides a mechanistic rationale for further evaluation of cadonilimab-based immunochemotherapy across different disease stages. Notably, the included studies employed heterogeneous chemotherapy backbones (i.e., FLOT, SOX, XELOX, and FOLFOX), which may influence the degree of synergy between dual checkpoint blockade and cytotoxic therapy. Different regimens can vary in their capacity to induce immunogenic tumor cell death and antigen release, thereby shaping antigen presentation and T-cell priming. In addition, the pooled pCR estimates observed in the neoadjuvant setting provide a rationale for continued evaluation of cadonilimab-based immunochemotherapy in the perioperative context.

Nevertheless, improvements in therapeutic efficacy are often accompanied by an increased risk of TRAEs. In the present analysis, grade 3 TRAEs were more frequently reported in the first-line advanced setting, with gastrointestinal toxicities, hematologic toxicities, and irAEs representing the most common categories. Although most irAEs were mild to moderate and generally manageable, close monitoring and timely intervention remain essential to minimize the risk of severe complications. In clinical practice, individualized risk stratification should consider baseline performance status, comorbidities, prior treatments, and immune status. A multidisciplinary approach may also help optimize safety and treatment adherence. Notably, our subgroup analyses suggested a lower incidence of grade 3 TRAEs in the neoadjuvant setting compared with first-line advanced treatment. This difference may partly reflect patient selection and baseline fitness (e.g., better organ reserve and fewer comorbidities), as well as a lower tumor burden and less cumulative treatment exposure in the neoadjuvant context. In addition, differences in chemotherapy backbone, dose intensity, and treatment duration across settings could contribute to divergent hematologic and gastrointestinal toxicity profiles. Practical factors, including monitoring intensity, reporting practices, and the limited sample size, may also have influenced the observed estimates. Therefore, these subgroup findings should be interpreted cautiously and warrant validation in larger prospective studies.

Several limitations of this meta-analysis warrant cautious interpretation. First, only four studies comprising 182 patients were included, and most were single-arm phase II trials without a control group, which limits causal inference regarding both efficacy and safety. Second, reporting of key endpoints was incomplete in some studies, particularly for pCR, irAEs, and OS; moreover, OS data were insufficient or unavailable in multiple studies, precluding quantitative pooling for this endpoint at present. In addition, differences in chemotherapy backbones, eligibility criteria, and timing of outcome assessment across studies may have contributed to variability in the pooled estimates. Although sensitivity analyses were performed to assess robustness, residual bias cannot be fully excluded. Therefore, larger randomized controlled trials with standardized endpoint reporting and longer follow-up are needed to clarify the clinical role of cadonilimab-based immunochemotherapy in G/GEJ adenocarcinoma, including the optimal treatment setting and patient selection. With additional high-quality evidence emerging, including survival outcomes reported by ongoing or future phase III trials, a future updated synthesis will be warranted to refine pooled estimates and confirm these findings.

## 5 Conclusion

This single-arm meta-analysis synthesized the currently available phase II evidence on cadonilimab combined with chemotherapy for patients with locally advanced or metastatic G/GEJ adenocarcinoma. The pooled estimates provide a quantitative summary of key efficacy outcomes, including ORR, DCR, pCR, and PFS, as well as the incidence of high-grade TRAEs. Overall, these findings suggest that cadonilimab plus chemotherapy shows clinical activity in G/GEJ adenocarcinoma, with a safety profile that appears manageable in the reported studies. Subgroup analyses suggested that safety profiles may differ between perioperative and advanced treatment settings; however, these findings should be interpreted cautiously given the limited number of included studies. Cadonilimab, a bispecific antibody targeting PD-1 and CTLA-4, represents a dual checkpoint blockade strategy that warrants further evaluation in G/GEJ adenocarcinoma. Nevertheless, the occurrence of irAEs highlights the importance of vigilant monitoring and appropriate patient selection in clinical practice. Given that only four early-phase studies were included and OS data were largely unavailable, larger randomized controlled trials with standardized endpoint reporting and longer follow-up are needed to validate these findings and better define the optimal treatment setting and patient population. As additional high-quality evidence becomes available, an updated quantitative synthesis will be warranted to refine and confirm these estimates.

## Data Availability

The original contributions presented in the study are included in the article/**Supplementary Material**. Further inquiries can be directed to the corresponding authors.
