# Supplementary figures and images for: Efficacy and safety of cadonilimab combined with chemotherapy for gastric or gastroesophageal junction adenocarcinoma: a single-arm meta-analysis
Source: Front Immunol. 2026 Feb 18;17:1693179. doi: 10.3389/fimmu.2026.1693179 (PMC12956804; doi:10.3389/fimmu.2026.1693179)

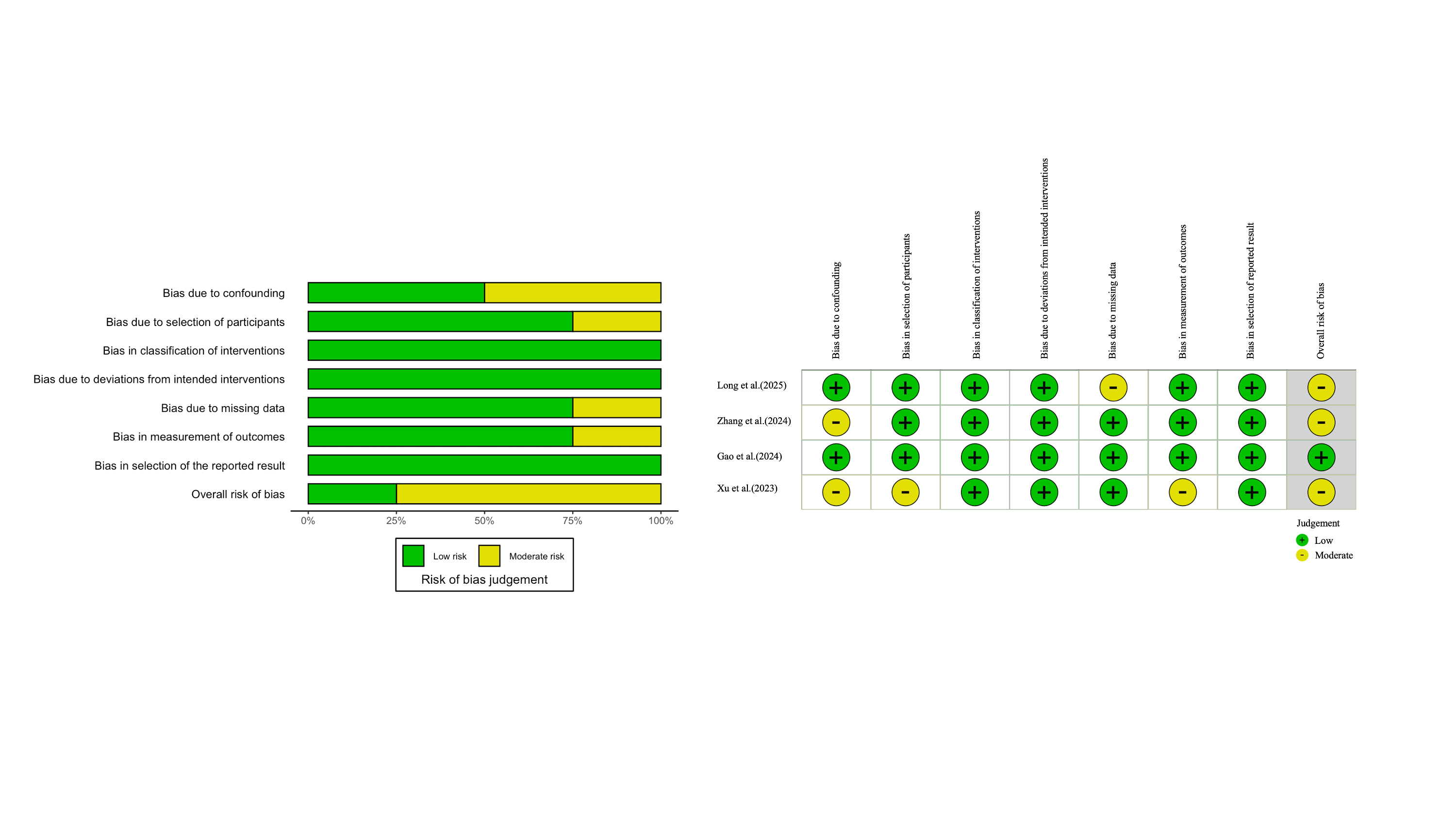

Supplement: Supplementary file 1 [file Image1.tiff]

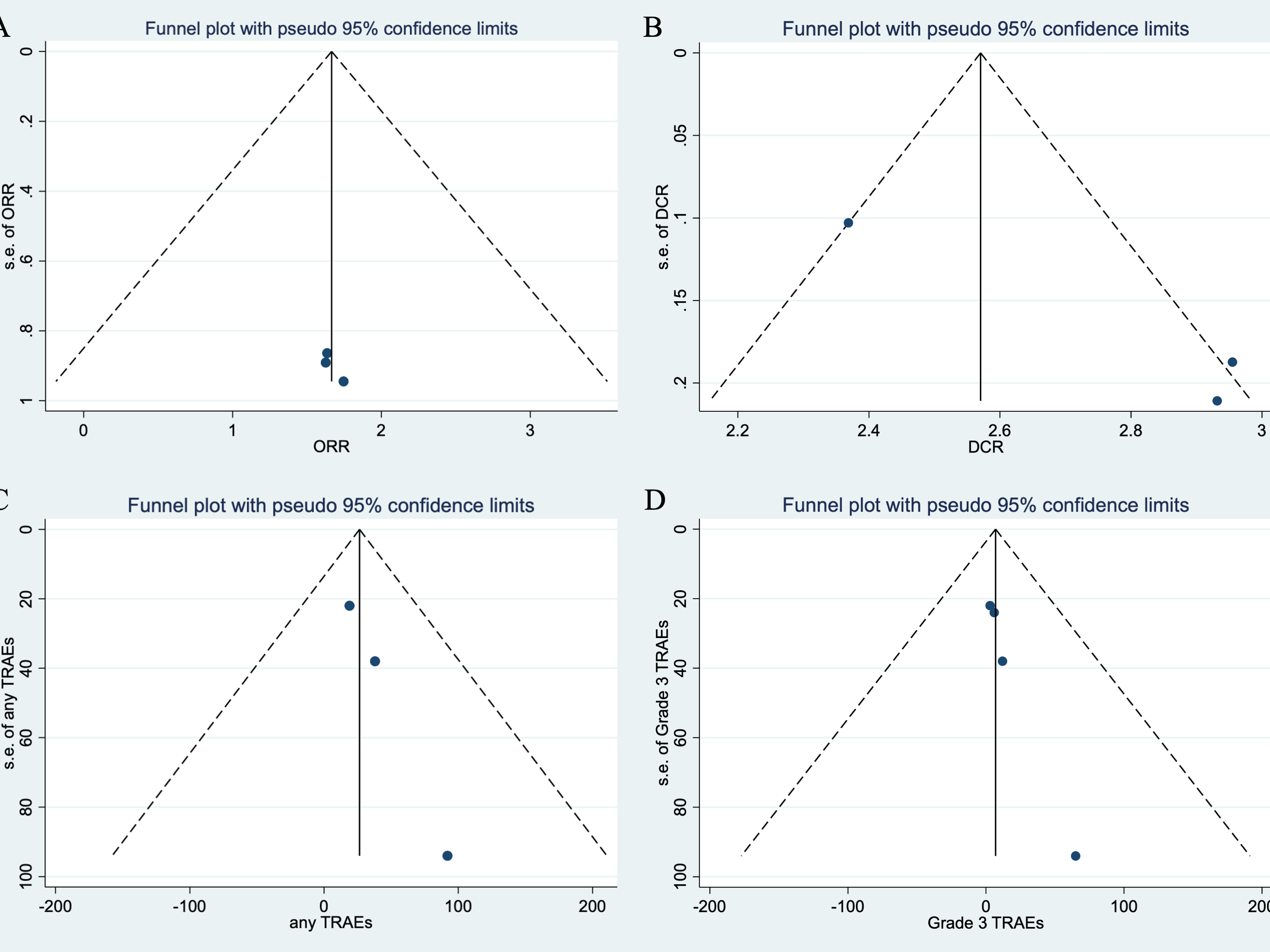

Supplement: Supplementary file 2 [file Image2.tiff]
