# Supplementary material for: Efficacy and safety of cadonilimab combined with chemotherapy for gastric or gastroesophageal junction adenocarcinoma: a single-arm meta-analysis
Source: Front Immunol. 2026 Feb 18;17:1693179. doi: 10.3389/fimmu.2026.1693179 (PMC12956804; doi:10.3389/fimmu.2026.1693179)
Supplement: Supplementary file 3 [file Table1.docx]

| Database | Search strategy |
| --- | --- |
| Pubmed | ((((((((((((((((((Stomach Neoplasms) OR (Neoplasm, Stomach)) OR (Stomach Neoplasm)) OR (Gastric Neoplasms)) OR (Gastric Neoplasm)) OR (Neoplasm, Gastric)) OR (Neoplasms, Gastric)) OR (Neoplasms, Stomach)) OR (Cancer of Stomach)) OR (Stomach Cancers)) OR (Cancer of the Stomach)) OR (Gastric Cancer)) OR (Cancer, Gastric)) OR (Cancers, Gastric)) OR (Gastric Cancers)) OR (Stomach Cancer)) OR (Cancers, Stomach)) OR (Cancer, Stomach) OR (Gastroesophageal Junction Adenocarcinoma) OR (Gastroesophageal Junction Cancer)) AND (((((((((cadonilimab) OR (AK104)) OR (Antibodies, Bispecific)) OR (Bifunctional Antibodies)) OR (Antibodies, Bifunctional)) OR (Bispecific Antibodies)) OR (Bispecific Monoclonal Antibodies)) OR (Antibodies, Bispecific Monoclonal)) OR (Monoclonal Antibodies, Bispecific)) |
| Web of Science | TS=("Gastric Cancer" OR "Stomach Cancer" OR "Gastric Neoplasm" OR "Stomach Neoplasm" OR "Cancer of Stomach" OR "Cancer of the Stomach") AND TS=("Cadonilimab" OR "AK104") |
| Embase | ('stomach cancer'/exp OR 'gastric cancer'/exp OR 'gastric carcinoma'/exp OR 'stomach neoplasm'/exp OR 'gastroesophageal junction cancer'/exp OR 'GEJ cancer'/exp) AND ('cadonilimab'/exp OR 'AK104' OR 'bispecific antibody'/exp OR 'bispecific antibodies'/exp OR 'bifunctional antibody'/exp OR 'bispecific monoclonal antibodies'/exp) |
| Cochrane Library | MeSH descriptor: [Stomach Neoplasms] explode all trees OR "Neoplasm, Stomach" OR "Stomach Neoplasm" OR "Gastric Neoplasms" OR "Gastric Neoplasm" OR "Neoplasm, Gastric" OR "Neoplasms, Gastric" OR "Neoplasms, Stomach" OR "Cancer of Stomach" OR "Stomach Cancers" OR "Cancer of the Stomach" OR "Gastric Cancer" OR "Cancer, Gastric" OR "Cancers, Gastric" OR "Gastric Cancers" OR "Stomach Cancer" OR "Cancers, Stomach" OR "Cancer, Stomach") AND "Cadonilimab" |
| ClinicalTrials.gov | (stomach cancer OR gastric cancer OR gastroesophageal junction cancer) AND (Cadonilimab OR AK104) |

Supplementary Table S1. Detailed search strategies for each database.
