# Supplementary material for: Efficacy and safety of cadonilimab combined with chemotherapy for gastric or gastroesophageal junction adenocarcinoma: a single-arm meta-analysis
Source: Front Immunol. 2026 Feb 18;17:1693179. doi: 10.3389/fimmu.2026.1693179 (PMC12956804; doi:10.3389/fimmu.2026.1693179)
Supplement: Supplementary file 4 [file Table2.docx]

| **Bias domain** | **Kappa value** | **95% CI** | **Agreement level** |
| --- | --- | --- | --- |
| Bias due to confounding | 0.5 | -0.235,1.000 | Moderate agreement |
| Bias in selection of participants | 1 | 1.000 to 1.000 | Almost perfect agreement |
| Bias in classification of interventions | 1 | 1.000 to 1.000 | Almost perfect agreement |
| Bias due to deviations from intended interventions | 1 | 1.000 to 1.000 | Almost perfect agreement |
| Bias due to missing data | 0.5 | -0.235,1.000 | Moderate agreement |
| Bias in measurement of outcomes | 1 | 1.000 to 1.000 | Almost perfect agreement |
| Bias in selection of reported result | 1 | 1.000 to 1.000 | Almost perfect agreement |
| Overall bias | 1 | 1.000 to 1.000 | Almost perfect agreement |

Supplementary Table S2. Inter-rater agreement for ROBINS-I risk of bias assessments using Cohen’s Kappa coefficient.
