# Supplementary material for: Efficacy and safety of cadonilimab combined with chemotherapy for gastric or gastroesophageal junction adenocarcinoma: a single-arm meta-analysis
Source: Front Immunol. 2026 Feb 18;17:1693179. doi: 10.3389/fimmu.2026.1693179 (PMC12956804; doi:10.3389/fimmu.2026.1693179)
Supplement: Supplementary file 5 [file Table3.docx]

| study | AE | Rate (%) | Event / Total |
| --- | --- | --- | --- |
| Long et al.(2025) | Constipation | 13.20% | 5/38 |
| Long et al.(2025) | Oral mucositis | 15.80% | 6/38 |
| Long et al.(2025) | Abnormal renal function | 2.60% | 1/38 |
| Long et al.(2025) | Proteinuria | 7.90% | 3/38 |
| Long et al.(2025) | Arrhythmia | 42.10% | 16/38 |
| Long et al.(2025) | Hypertension | 63.20% | 24/38 |
| Long et al.(2025) | Cardiac insufficiency | 31.60% | 12/38 |
| Long et al.(2025) | Headache | 21.10% | 8/38 |
| Long et al.(2025) | Peripheral neuritis | 39.50% | 15/38 |
| Long et al.(2025) | Hoarseness | 5.30% | 2/38 |
| Long et al.(2025) | Dysesthesia | 2.60% | 1/38 |
| Long et al.(2025) | Body pain | 5.30% | 2/38 |
| Long et al.(2025) | Night sweat | 13.20% | 5/38 |
| Long et al.(2025) | Dyspnea | 5.30% | 2/38 |
| Long et al.(2025) | Edema | 15.80% | 6/38 |
| Long et al.(2025) | Electrolyte disturbance | 78.90% | 30/38 |
| Long et al.(2025) | Alopecia | 94.70% | 36/38 |
| Zhang et al.(2024) | dermatitis | 29.20% | 7/24 |
| Gao et al.(2024) | Asthenia | 24.50% | 23/94 |
| Gao et al.(2024) | Infusion-related reaction | 23.40% | 22/94 |
| Gao et al.(2024) | Amylase increased | 21.30% | 20/94 |
| Gao et al.(2024) | Decreased appetite | 17.00% | 16/94 |
| Gao et al.(2024) | Blood bilirubin increased | 16.00% | 15/94 |
| Gao et al.(2024) | Hypoesthesia | 16.00% | 15/94 |

Supplementary Table S3. TRAEs reported in single studies only, with corresponding incidence rates.
